# Predictors of Treatment Response to an Internet-Delivered Intervention Targeting Residual Cognitive Symptoms After Major Depressive Disorder

**DOI:** 10.3389/fpsyt.2022.795698

**Published:** 2022-03-28

**Authors:** Sunniva Brurok Myklebost, Rolf Gjestad, Yavuz Inal, Åsa Hammar, Tine Nordgreen

**Affiliations:** ^1^Department of Biological and Medical Psychology, Faculty of Psychology, University of Bergen, Bergen, Norway; ^2^Division of Psychiatry, Haukeland University Hospital, Bergen, Norway; ^3^Department of Design, Faculty of Architecture and Design, Norwegian University of Science and Technology, Gjøvik, Norway; ^4^Department of Global Public Health and Primary Care, Faculty of Medicine, University of Bergen, Bergen, Norway

**Keywords:** depression, internet-based, cognitive enhancement therapy, cognitive remediation, web-based, cognitive impairment

## Abstract

**Objective:**

Residual cognitive symptoms after depression are common and associated with reduced daily life functioning and an increased risk of depression relapse. There is a lack of knowledge on treatments targeting residual cognitive symptoms after major depressive disorder (MDD), including the factors associated with treatment response. The aim of the current study is to explore factors of treatment response to a guided internet-delivered intervention for former depressed adults experiencing residual cognitive symptoms.

**Method:**

Forty-three former depressed adults with residual cognitive symptoms were included. Linear mixed model analyses were used to investigate the impact of pre-treatment demographic-, illness, and symptom variables, and therapy process variables, such as credibility, expectancy, and user behavior, on reduction in residual cognitive symptoms from pre-treatment to 6-month follow-up.

**Results:**

Having had MDD for a year or less predicted more reductions in residual cognitive symptoms from pre- to 6-month follow-up. Higher levels of perceived treatment credibility and expectancy evaluated in the early course of treatment did also predict a positive treatment response. No demographic-, symptom-variables, previous number of episodes with MDD, and user behavior were associated with change in residual cognitive symptoms.

**Conclusion:**

This study suggests that individuals with shorter duration of previous depressions might have larger reductions in residual cognitive symptoms at 6-month follow-up compared to those with a longer duration of depression. Treatment credibility and expectancy also predicted treatment response and effort should also be made to ensure interventions credibility. Results should be interpreted with caution due to the study having a low sample size. Further investigation of predictors should be conducted in a full scale randomized controlled trial.

## Introduction

Major depressive disorder (MDD) is a leading cause for the burden of disease with high prevalence and a recurring course (1, 2). Although mood symptoms are the most prominent feature of MDD, cognitive functions are also affected (3). More specifically, reduced cognitive functions related to attention, memory, and executive domains are recognized as core symptoms in acute states of MDD (4). Clinical research and practice have also documented cognitive difficulties to persist as residual symptoms in states of remission and as a consequence associated with poor everyday functioning, quality of life, and an increased probability of depression relapse (5–7). In order to reduce the negative consequences and to increase functioning after MDD, residual cognitive symptoms are emerging as an important treatment target after MDD. Clinical trials evaluating the effects of interventions targeting residual cognitive symptoms after MDD do show promising results in face-to-face (8) and internet-delivered (9, 10) formats on working memory, attention, planning, long-term verbal memory, switching abilities, in addition to psychosocial functioning, brooding, and emotion regulation strategies. In a recent study, we reported large reductions in residual cognitive symptoms (*d* = 1.06) from using the internet for delivering a novel intervention for residual cognitive symptoms after MDD (11). However, little is known about the predictors associated with treatment response from interventions targeting residual cognitive symptoms after MDD.

Identifying predictors of treatment outcome are of importance to make clinical decisions such as finding the treatment most appropriate to which individuals, in addition to refining and tailoring treatment in order to meet the needs of those not responding (12, 13). This has implication for clinical guidelines, treatment optimisation, cost-effectiveness, and use of time resources that are relevant to implementation into routine care. However, there are only a few previous studies reporting on predictors associated with treatment response from interventions targeting cognitive difficulties related to MDD. First, a recent RCT study of mildly depressed adults receiving a face-to-face intervention targeting cognitive difficulties did not identify any sociodemographic, cognitive, illness-related, or psychological predictors of treatment response receiving a face-to-face intervention targeting cognitive difficulties (14). Another intervention study targeting cognitive impairment after MDD explored similar variables and found that those individuals improving their attention after treatment had a history of being depressed for fewer years than individuals not showing improvement (15). Indicating that duration of depression may be a predictor of treatment outcome. Studies are thus needed to evaluate if duration of depression is a consistent predictor of treatment response in interventions targeting residual cognitive symptoms. To our knowledge, no studies have explored predictors for treatment response of an internet intervention targeting residual cognitive symptoms after MDD.

In the search for predictors for internet-delivered interventions for MDD both demographic and baseline primary symptoms have been explored. However, results are mostly inconclusive as some studies find that these variables predict treatment response while others have not (16). Thus, higher pre-treatment levels of depression and being female have been shown to predict treatment response in internet-delivered interventions for depression (17–21). However, more recent studies indicates that data collected during the course of treatment may be more effective in predicting treatment outcome than pre-treatment data (22). Results from these studies do show that perceived treatment credibility and expectancy measured in the early course of treatment predicts drop-out, adherence, and positive treatment outcome (23, 24). Another promising approach may be the use of predictors related to the therapy process itself, such as user-behavior. Exposure to internet interventions, measured by higher number of logins, average time used in each session, and number of activities completed in each session, have all been reported as associated with a decrease in depression symptoms (25, 26). Data regarding usage of interventions are easily accessible in internet-delivered interventions and therefore provide opportunities to gain insights into the role of user behavior predicting treatment response.

The aim of the current study is to explore predictors of change in residual cognitive symptoms from pre- to 6-month follow up after receiving a guided internet-delivered treatment for former depressed adults (11). More specifically, we explored the following variables based on data collected pre-treatment: [1] demographic variables (age, gender, education, civil status); [2] depression history variables (episodes of depression, duration of depression); [3] symptom variables (depression load, rumination, self-efficacy), and the following variables measured during treatment; [4] treatment credibility and expectancy; [5] user-behavior (number of logins, session length, days in program).

## Methods

The present study was approved by the Regional Committee for Medical Research Ethics of Western Norway (2018/2384/REK vest). Data analyzed in the study were collected for an exploratory open trial. Informed consent was obtained from all participants.

### Design, Participants, and Procedures

This study is a secondary analysis of an open trial (11). We have in the current study also used data collected during treatment. The original trial was explorative where the aim was to investigate change in clinical outcomes including residual cognitive symptoms, rumination, and depression symptoms at post-treatment and 6-month follow-up that would inform a later large-scale trial. A total of 43 former depressed adults was included in the orginal trial. Results from linear mixed model analyses showed large reductions in residual cognitive symptoms (*d* = 1.06), rumination (*d* = 0.86), and no change in depression symptoms. Among the participants that completed the intervention did 55% show a reliable improvement from pre-treatment to 6-month follow-up, 42% were unchanged, and 3% deteriorated. Data was collected between March 2019 and May 2020. Participants were self-recruited through social media, posters, and traditional media. The inclusion criteria were the same for the primary and secondary analyses where participants needed to: (a) previously received treatment for MDD in primary or secondary healthcare services; (b) have few or minor depression symptoms with no cardinal symptoms reported sadness, loss of interest, and inability to feel; <16 on the Montgomery-Åsberg Depression Rating Scale [MADRS; (27)]; (c) self-report residual cognitive symptoms that affected daily functioning; (d) have no changes in psychopharmacological treatment under the study period; (e) be between 18 and 65 years, and (f) have internet access. The following exclusion criteria were used: (a) self-reported substance abuse; (b) neurological conditions or damage (e.g. autism, cerebral hemorrhage, and brain tumor); (c) bipolar disorder; and (d) psychosis. Interested adults contacted the research team and were assessed via telephone for eligibility by a clinical psychologist or an advanced student in clinical psychology. The M.I.N.I. Norwegian version, a structural clinical interview for psychiatric diagnoses in the DSM-IV (28) was used to confirm previous depression diagnosis and absence of current depression. Those participants found eligible responded to an additional online assessment that included The Montgomery-Åsberg Depression Rating Scale Self-report [MADRS-S; (29)] and open-ended questionnaires about their experience with residual cognitive symptoms. Among the 43 individuals that were included in the study did six participants have missing data at post-assessment and 11 did not respond to the 6-month follow-up assessment.

### Intervention

The user-centered development of the intervention is described in a previous paper (30). The intervention was internet-delivered and based on key components from cognitive enhancement therapy for mood disorders (31) including psychoeducation, cognitive training, strategy training, and tasks to enhance the transfer of skills into daily life as illustrated in Table 1. The participants were provided with ten intervention modules and encouraged to complete them within 5–7 weeks. Access to the intervention material was given through a secure digital platform and content was delivered in the combination of text, videos, photos, and audio recordings. Weekly telephone guidance was given during treatment by a clinical psychologist or an advanced student in clinical psychology receiving weekly supervision by a senior clinical psychologist. The therapist support focused on goal setting, goal attainment, explaining intervention material, and monitoring depression symptoms such as suicidal thoughts. Participants had access to the intervention material in the follow-up period but did not receive therapist support. All participants were offered an optional telephone conversation at the 6-month follow-up.

**Table 1:** Overview of intervention key components, intervention elements, and presumed mechanisms of change.

**Key components**	**Intervention elements**	**Presumed mechanisms of change**
Psychoeducation	• Psychoeducation on residual cognitive symptoms, rumination, and rationale of treatment.	Insight, hope, normalization, engagement, and credibility.
Cognitive activation	• Psychoeducation about the role of attention and training on different aspects of attention in daily life.	Cognitive control and flexible shifting attention.
Strategy training	Strategies to cope with attention difficulties: • Self-talk during task completion. • Taking frequent breaks. • Reducing distractions in surroundings. Strategies to cope with memory difficulties: • External aids (e.g. calendar, notes). • Rephrasing information. • Mnemonic training. Strategies to cope with executive difficulties: • Organizing home environment. • Daily activity planning. • Stepwise problem-solving model. Strategies to cope with rumination and worries: • Registration of negative thoughts and helpful thoughts related to cognitive difficulties. • Scheduling for rumination and worries. • Gratitude exercise. Strategies for social cognition: • Active listening and keeping eye contact. • Rephrasing messages.	Sustained attention. Prospective memory and information retrieval. Initiation of activities and problem-solving skills. Reduce rumination and worries that occupy cognitive resources. Conversational attention.
Self-tailoring and transfer to daily life	• Workbook “My plan” where participants can freely select and register training tasks. Registered tasks are tested in a daily life context and evaluated.	Motivation and transfer skills to daily life.

### Outcome Measure

The Behavior Rating Inventory of Executive Function-Adult version [*BRIEF-A*; (32)] was employed to measure the level and change in residual cognitive symptoms. The BRIEF-A is a 75-items questionnaire measuring adults' self-reported executive difficulties in everyday life. Individuals respond to each item according to frequency as either 1 (never), 2 (sometimes) and 3 (often). The results can be comprised into nine clinical subscales: Inhibit, Shift, Self-monitor, Emotional control, Initiate, Working memory, Plan/Organize, Task monitor, and Organization of materials. These clinical sub-scales can be incorporated into a summary score, the BRIEF-A Global Executive Composite (BRIEF-A GEC). Higher scores on the BRIEF-A GEC indicate more executive problems. Participants responded to the BRIEF-A at pre-treatment, post-treatment, and 6-month follow-up. The study used BRIEF-A raw scores to analyze significant change. However, T-scores may also be calculated, where a T-score > 65 is considered a threshold for clinically significant executive difficulties. Thus, there are no Norwegian norms for BRIEF-A and studies indicate that the American norms underestimates executive difficulties in Norwegian samples (33). Therefore, may not a change to under the clinical threshold of 65 be appropriate as a measure of clinical improvement for the sample in the current study. In the original study (11) were participants showing a reduction on the reliable change index score of 14 points or more categorized as improvers. This is comparable to another recent study defining 13 points reduction on the BRIEF-A GEC as a reliable improvement (14). The BRIEF-A has been validated and demonstrated good internal consistency in previous studies (11, 32, 34).

### Predictor Variables

No a priori hypotheses were formulated before the analyses as the aim was to explore predictors of a novel intervention. Thus, based on the literature potential predictors were included in the study protocol. These predictors were explored in the preliminary analyses and based on this we included predictors related to the following: demographic, illness history, pre-treatment symptoms, and therapy process.

#### Demographic Variables

Demographic information was collected before treatment started. Included in the current analysis are age, gender, civil status, and education.

#### Illness History Variables

Data on depression history were collected pre-treatment. The duration of depression was analyzed as being depressed for one year or less, or longer than one year. The number of depression episodes were analyzed as either one episode of depression or several episodes of depression.

#### Pre-treatment Symptom Variables

Symptom variables were collected pre-treatment and included level of depression load, rumination, and self-efficacy.

Levels of depression load at pre-treatment were measured using the MADRS-S which consists of nine items. The sum of the scores for the nine items ranges from 0 to 54 where higher scores indicate higher depression load. The online version of MADRS-S has demonstrated good psychometric properties (35).

To measure the pre-treatment level of rumination the Rumination Response Scale [RRS; (36)] was used. The RRS is a 22-item four-point Likert scale questionnaire that assesses the degree of ruminative responses to depressed mood. Each item is rated from 1 (almost never) to 4 (almost always) resulting in a total score ranging from 22 to 88, in which a higher score indicates higher levels of rumination (37). The RRS have demonstrated good internal consistency and have been validated in previous studies (38, 39).

The pre-treatment level of self- efficacy was measured using the General Perceived Self-efficacy Scale [GSE; (40)], which comprises 10 items scored 1 (Not at all true) to 4 (Exactly true). Examples of the items are “I can solve most problems if I invest the necessary effort”; or “I can remain calm when facing difficulties because I can rely on my coping abilities.” Total scores range between 10 and 40, with a higher score indicating more self-efficacy. The GSE has demonstrated good psychometric properties (41).

#### Therapy Process Variables

Therapy process variables were based on data collected during treatment and included treatment credibility and expectancy, and user behavior (number of logged on sessions, average length of sessions, and days in treatment).

Treatment credibility and expectancy was measured after completion of the first module employing a modified version of the Credibility and Expectancy Scale (CEQ) comprising 5 items (42). The participants rated perceived treatment credibility on a scale from 0 to 10 where higher scores indicated better treatment credibility. Examples of the items are “How much sense does the therapy offered to you seem to make?” and “How much improvement do you expect to get after treatment?”

Data regarding usage of the intervention were stored in the digital platform delivering the intervention. The total number of logged on sessions during the intervention period was collected, and the average length of sessions were calculated by dividing the number of minutes in the interventions by the number of occasions they had logged on to the program. Days in treatment were calculated based on the dates between the first login into the intervention and the completion of tasks in module 10.

### Statistical Analyses

Descriptive analyses and linear mixed model analyses were performed using SPSS statistical software package version 25. The threshold for statistical significance was set to p <0.05. Linear mixed model analyses were used to analyse the impact of the predictor variables, one at a time, on pre-treatment level and change scores in the BRIEF-A GEC from pre-treatment to 6-month follow-up. The linear mixed model was specified as a random intercept fixed slope model and the estimator was set to restricted maximum likelihood. Only fixed effects are presented. Missing values were assumed to be missing at random (43) and all available information in outcome for participants were included in the analyses. In order to enhance interpretation of the results continuous predictor variables were mean centered. A Bonferroni *post hoc* test was used to adjust for multiple tests (*p* < 0.004). In Tables 2, 3 in the result section are variables that were significant after Bonferroni correction are presented in bold. No co-variates were included in the analyses.

**Table 2:** Linear mixed model with estimated pre-treatment level and change in residual cognitive symptoms (BRIEF-A GEC) adjusted for categorical demographic, illness history, and therapy process variables (*N* = 43).

**Variables**	**Pre-treatment level (95% CI)**	***p*-value**	**Change (95% CI)**	***p*-value**
**Demographic**				
Male	137.33 (123.07–151.60)	0.632	−28.44 (−42.54 to −14.34)	0.198
Female	−3.86 (−19.90–12.18)		10.36 (−5.55–26.26)	
Partner	129.00 (119.55–138.44)	0.132	−14.58 (−23.15 to −6.01)	0.051
No partner	9.87 (−3.05–22.79)		−12.93 (−25.90–0.05)	
Higher education	135.07 (127.17–142.96)	0.362	−20.23 (−27.96 to −12.32)	0.974
No higher education	−2.61 (−16.96–11.75)		−0.24 (−14.66–14.19)	
**Illness history**				
≤1 year	135.10 (121.79–148.41)	0.889	−38.34 (−50.60 to −26.09)	**0.001**
>1 years	−1.07 (−16.26–14.12)		24.30 (10.18–38.41)	
1 episode	131.08 (118.75–143.42)	0.545	−20.92 (−31.98–9.85)	0.874
≥2 episodes	4.43 (−10.09–18.96)		1.09 (−12.64–14.82)	

*Bold indicates that the p-value is significant after Bonferroni correction; Pre-treatment levels: the intercept on the BRIEF-A GEC and the estimated difference on the BRIEF-A GEC between predictor categories at pre-treatment; Change: change from pre-treatment assessment to 6-month follow-up on the BRIEF A GEC and the difference in BRIEF-A GEC scores between predictor categories from pre-treatment to 6-month follow-up; BRIEF-A GEC, The Behavior Rating Inventory of Executive Function-Adult Global Executive Composite; CI, confidence interval*.

**Table 3:** Linear mixed model with estimated intercept levels for pre-treatment level and change, pre-treatment levels, and change in residual cognitive symptoms (BRIEF-A GEC) adjusted for continuous demographic, symptom, and therapy process variables (*N* = 43).

	**Intercept levels**		**Prediction of pre-treatment levels and change**			
**Variables**	**Pre-treatment (95% CI)**	**Change (95% CI)**	**Pre-treatment (95% CI)**	***p*-value**	**Change (95% CI)**	***p*-value**
**Demographic**						
Age	134.28 (127.76–140.80)	−20.60 (0.27.12 to −12.09)	−0.18 (−0.68–0.33)	0.492	0.18 (−0.30–0.67)	0.455
**Symptom**						
RRS	134.28 (128.33–140.23)	−20.76 (−27.32 to −14.19)	0.50 (−0.01–1.01)	0.056	0.31 (−0.24–0.87)	0.259
MADRS-S	134.28 (127.72–140.83)	−20.10 (−26.70 to −13.51)	0.89 (−1.15–2.94)	0.387	−0.64 (−2.85–1.56)	0.562
GSE	134.27 (129.10–140.44)	−20.08 (−26.66 to −13.50)	−2.13 (−4.01 to −0.25)	0.027	−0.02 (−1.02–1.97)	0.981
**Therapy process**						
CEQ	134.45 (128.33–140.58)	−17.38 (−24.11 to −10.67)	−0.38 (−1.23–0.46)	0.370	−1.71 (2.91 to −0.51)	0.006
Session's total	134.27 (128.02–140.54)	−20.47 (27.08 to −13.85)	−0.78 (−1.50 to −0.07)	0.031	0.37 (−0.37–1.10)	0.324
Session length	134.29 (127.69–140.88)	−20.13 (−26.69 to −13.57)	0.14 (−0.22–0.50)	0.441	−0.17 (−0.55–0.20)	0.353
Days in treatment	134.28 (127.79–140.77)	−20.07 (−26.60 to −13.54)	−0.08 (−0.43–0.27)	0.661	0.43 (0.12–0.98)	0.125

*Intercept levels Pre-treatment: estimated pre-treatment levels on the BRIEF-A GEC when predictor variables are centered; Intercept levels Change: estimated level of change in the BRIEF-A GEC when the effect of the predictor variable is centered; Prediction of pre-treatment level: estimated level in the BRIEF-A GEC per unit change in predictor variable at pre-treatment; Prediction and change: difference scores in the BRIEF-A GEC per unit change on the predictor variable from pre-treatment to 6-month follow-up. BRIEF-A GEC, The Behavior Rating Inventory of Executive Function-Adult Global Executive Composite; RRS, rumination response scale; MADRS-S, Montgomery Åsberg Depression Rating Scale Self-report; GSE, General self-efficacy scale; CEQ, credibility and expectancy questionnaire; CI, confidence interval*.

## Results

In the current paper we explored predictors of treatment response from pre-treatment to 6-month follow-up. The following variables was explored: [1] demographic variables (age, gender, education, civil status); [2] depression history variables (episodes of depression, duration of depression); [3] symptom variables (depression load, rumination, self-efficacy), and the following variables measured during treatment; [4] treatment credibility and expectancy; [5] user-behavior (number of logins, session length, days in program).

Participant characteristics are presented in Table 4.

**Table 4:** Characteristics of the study sample (*N* = 43).

**Sample characteristics**	**n (%)/M**	**SD**
Age	35.28	(13.04)
**Gender**
Male	9 (21%)	
Female	34 (79%)	
**Educational level**
Higher education	30 (70%)	
No higher education	13 (30%)	
**Civil status**
Partner	20 (47%)	
No partner	23 (53%)	
Antidepressant use*
No	29 (67%)	
Yes	14 (33%)	
**Duration of depression**
≤1 year	10 (23%)	
>1 year	33 (77%)	
**Depression episodes**
≤1 episode	12 (30%)	
≥2 episodes	31 (72%)	
**Symptom variables**
Cognitive symptoms (BRIEF-A)	134.28	19.06
Rumination (RRS)	42.65	11.80
Depression load (MADRS-S)	8.74	3.24
Self-efficacy (GSE)	25.05	3.32
**Therapy process variables**
Treatment credibility and expectancy (CEQ)	36.45	7.31
Session's total	18.88	8.96
Session length	40.45	18.48
Days in treatment	42.16	18.79

n, number of participants with the specified characteristic; M, mean values for the specified characteristics; SD, standard deviation; BRIEF-A GEC, The Behavior Rating Inventory of Executive Function-Adult Global Executive Composite; RRS, rumination response scale; antidepressant use*, antidepressant use the last two years; MADRS-S, Montgomery Åsberg Depression Rating Scale Self-report; GSE, General self-efficacy scale; CEQ, Credibility and expectancy questionnaire; Session total, number of times logged onto the intervention; Session length, average number of minutes for each session.

### Differences in Pre-treatment BRIEF-A GEC Scores Across Demographic, Depression History, Symptom-, and Therapy Process Variables

Results from linear mixed model analyses included investigation of pre-treatment differences in BRIEF-A GEC scores within categorical variables (Table 2) and in continuous variables (Table 3).

Within the demographic variables there were no significant differences in BRIEF-A GEC pre-treatment scores. No significant difference on pre-treatment levels on the BRIEF-A GEC were obtained within the illness history categories. Among the symptom variables were self-efficacy levels at pre-treatment significantly associated with pre-treatment levels on the BRIEF-A GEC, which show that participants with initial higher levels of self-efficacy had lower scores on the BRIEF-A GEC before treatment started. Rumination levels at pre-treatment were almost significant, showing a tendency that participants with initial high levels of rumination having higher scores on the BRIEF-A GEC at pre-treatment. No significant difference on pre-treatment levels of depression symptoms were observed.

Among the therapy process variables, based on user-data, were pre-treatment levels on the BRIEF-A GEC associated with the number of logged sessions during the intervention period. This indicates that individuals with higher levels of logged sessions had lower scores on the BRIEF-A GEC before treatment.

The *post hoc* Bonferroni test showed that none of the variables were significantly associated with pre-treatment differences on the BRIEF-A GEC.

### Impact of Demographic-, Depression History, Symptom-, and Therapy Process Variables on BRIEF-A GEC Change Scores

Results from linear mixed model analyses are presented in Table 2 for categorical variables and Table 3 for continuous variables.

None of the demographic variables showed significant impact on the BRIEF-A GEC change scores from pre-treatment to 6-month follow-up. Thus, there were a tendency for those without a partner to have larger reductions on the BRIEF-A GEC at follow-up compared to individuals having a partner. Duration of depression for one year or less did predict larger reductions on the BRIEF-A GEC compared to participants with longer duration of depression. Number of previous depression episodes did not have a significant impact on BRIEF-A GEC change scores. Symptom variables that included pre-treatment levels of depression load, rumination, and self-efficacy did not predict change scores on the BRIEF-A GEC.

Among the therapy process variables, based on data collected during treatment, treatment credibility and expectancy significantly predicted change in the BRIEF-A GEC, showing that participants with higher levels of treatment credibility had larger reductions on the BRIEF-A GEC from pre-treatment to follow-up.

The *post hoc* Bonferroni test taking into account all analyses showed that only duration of depression was significantly related to treatment response.

## Discussion

This study explored predictors associated with change in residual cognitive symptoms at 6-month follow-up after receiving an internet-delivered intervention for former depressed adults. Key findings from the current study were that a history of being depressed for less than a year predicted larger reductions in residual cognitive symptoms compared to those with longer duration of depression. Another main finding from the study was that high levels of perceived credibility and expectancy were associated with a change in residual cognitive symptoms. We did not find any of the demographic variables that included age, gender, education level, and civil status to predict treatment response. Neither did any of the symptom's variables such as rumination, depression symptoms, or self-efficacy. We did also not find that previously being depressed once or several times impacted treatment response. Participants' usage of the intervention did also not predict treatment outcome. Overall, these findings must be interpreted with caution due to the study having a low sample size and many predictors.

In line with a previous study, we found that the duration of depression was a significant predictor of treatment response, whereas number of depressive episodes were not (15). Shorter illness duration has also been found to be associated with better treatment outcome following interventions targeting cognitive difficulties in schizophrenia (44, 45). This might be explained by cognitive difficulties worsening during the course of depression due to neurobiological scarring that persists in remission (15). Individuals with a history of longer periods of depression might also be more vulnerable to experiencing mood symptoms under treatment that further influence cognitive difficulties and consequently affect treatment response (46). However, it is important to note that also those participants in the current study having been depressed for longer periods also showed reductions in residual cognitive symptoms, although not as large as those being depressed for less than a year.

Rumination measured before treatment did not predict treatment response, in line with another study (14). This result may indicate that higher pre-treatment levels of rumination are not a barrier for gaining reduction in cognitive difficulties compared to those with lower levels of symptoms. Moreover, in the current study pre-treatment depressive symptoms were not associated with treatment outcome. This is also in line with previous studies on interventions targeting cognitive difficulties in depression (14, 15). This could be explained by the fact that moderate and high levels of depressive symptoms were an exclusion criteria in the current sample, and consequently making the level of depression symptoms low, with insufficient variability to detect significant individual differences. Furthermore, pre-treatment levels of self-efficacy were not significantly associated with treatment response, which is in accordance with studies on internet interventions for depression (16). The interplay between the intervention and self-efficacy is however not explored in the current study. The intervention itself may have an impact on self-efficacy levels, which might mediate treatment response. Future studies may therefore explore the role of self-efficacy levels during therapy in predicting treatment outcome.

Perceived intervention credibility and expectancy did predict change in residual cognitive symptoms which is consistent with studies reporting patients' beliefs in and trust toward interventions to be related to treatment response (47–49). The current intervention might have improved credibility levels as it included elements known to ensure credibility, such as providing a clear rationale of symptoms from credible sources, self-tailoring, and an easy-to-use digital infrastructure (50). However, the study sample was self-referred to the intervention and the role of treatment credibility may be different in a routine care setting.

Contrary to previous research reporting exposure to intervention material being related to improvement of depression symptoms (26), we did not find that number of logins, average session length, and days in treatment did predict treatment response. However, we have not used data on participants' number of activities for each logged session which in previous research has been found to be a predictor of treatment response in an intervention targeting depression symptoms (25). Consequently, the role of user behavior as a predictor of treatment response in interventions targeting residual cognitive symptoms needs further exploration.

### Clinical Implications

The clinical implications of the findings from the present study must be regarded as preliminary due to low sample size. However, these exploratory analyses may indicate that reaching individuals with interventions targeting cognitive difficulties in their first year of depression should be considered in future implementation into routine care. Nevertheless, participants being depressed for over a year also gained reductions in residual cognitive symptoms in the present study and this group may therefore also benefit from receiving treatment. Participant's perceptions of the intervention as being credible may be of relevance to treatment response. Effort may therefore be made to ensure the credibility of the intervention in a routine care setting. Lastly, future studies employing theory and evidence-based approaches to investigate predictors of treatment response in similar interventions may be informed by the results of this study.

### Strengths and Limitations

The study has several limitations that need to be addressed. The study design was an open trial, with an unblinded outcome measure, and analyses were exploratory making conclusions tentative. Moreover, the robustness and generalizability of the results are reduced by the small sample size and many predictors included. Also the small sample size increases the risk of type II errors and some variables in this study may have been significant related to outcome with a larger sample size. Still, the duration of depression impacting treatment response is in line with previous research on the same target group reporting similar results (15). The results of credibility and expectancy as a predictor of treatment outcome is thus more uncertain as this showed not to be significant after the Bonferroni correction and should therefore be explored further. Employing the linear mixed model approach is a strength in studies with a small sample size as all available data are included in the analyses consequently affecting power. Therefore, we do not have to assume missing completely at random (MCAR) but missing at random (MAR). However, as is the case in most intervention studies we cannot be sure if missing data are missing not at random (MNAR), as this hypothesis is not empirical testable. However, several of the above limitations are to be addressed in a currently ongoing randomized controlled trial with a larger sample size (ClinicalTrials.gov: 04864353). The impact of current and previous treatment was not explored. Among the participants had 33% received treatment with antidepressant drugs the last two years. This may have impacted treatment outcome as previous studies shows antidepressant drugs to relief cognitive symptoms during depression (51), thus also being associated with side effects such as cognitive difficulties during remission from MDD (52). Investigating the effects of combining antidepressant treatment with the intervention may therefore be a future research target. The main strength of the current study includes analyzing follow-up assessments that provides knowledge about predictors of long-term treatment response. Another strength is the novel approach of investigating the role of participants usage of the intervention in predicting treatment response.

## Conclusion

This study suggests that individuals with shorter duration of previous depressions might have larger reductions in residual cognitive symptoms at 6-month follow-up compared to those with a longer duration of depression. Treatment credibility and expectancy also predicted treatment response and effort should also be made to ensure interventions credibility. An important next step will be to investigate the role of these predictor variables based on data from full scale randomized controlled trials.

## Data Availability Statement

The raw data supporting the conclusions of this article will be made available by the authors, without undue reservation.

## Ethics Statement

The studies involving human participants were reviewed and approved by Regional Committee for Medical and Health Research Ethics Western Norway. The patients/participants provided their written informed consent to participate in this study.

## Author Contributions

SM collected the data and wrote the manuscript draft. SM and YI organized and calculated the user-data. SM, TN, and RG participated in the analyses and interpretation of data. TN acted as main supervisor for this study, which was a part of a doctoral thesis, and contributed with several revisions of the manuscript draft. ÅH contributed with a conceptualization of the original trial and revisions of the manuscript draft. YI and RG contributed to the revision of the manuscript draft. All authors contributed to the article and approved the submitted version.

## Funding

This study is part of the Introducing Mental health through Adaptive Technology funded by the Norwegian Research Council (NFR: 259293).

## Conflict of Interest

The authors declare that the research was conducted in the absence of any commercial or financial relationships that could be construed as a potential conflict of interest.

## Publisher's Note

All claims expressed in this article are solely those of the authors and do not necessarily represent those of their affiliated organizations, or those of the publisher, the editors and the reviewers. Any product that may be evaluated in this article, or claim that may be made by its manufacturer, is not guaranteed or endorsed by the publisher.
